# A higher preoperative total protein to albumin ratio independently predicted more severe postoperative acute kidney injury in patients with acute type A aortic dissection: a retrospective cohort study of 224 cases

**DOI:** 10.3389/fcvm.2025.1562388

**Published:** 2025-05-12

**Authors:** Xiuhua Wang, Hui Xu, Guowei Tu, Hao Lai, Jiarui Xu, Xin Li, Zhe Luo

**Affiliations:** ^1^ Department of Cardiac Surgery, Zhongshan Hospital, Fudan University, Shanghai, China; ^2^ Cardiac Intensive Care Center, Zhongshan Hospital, Fudan University, Shanghai, China; ^3^ Department of Nephrology, Zhongshan Hospital, Fudan University, Shanghai, China

**Keywords:** total protein to albumin ratio, acute kidney injury, risk factors, predictive model, total aortic arch replacement surgery

## Abstract

**Objectives:**

In this retrospective study, we investigated the incidence of postoperative acute kidney injury (AKI) and determined the predictors associated with AKI in patients underwent surgeries for acute type A aortic dissection (ATAAD).

**Methods:**

We enrolled patients diagnosed with ATAAD and received operation. AKI was defined based on the Kidney Disease: Improving Global Outcomes criteria. Potential perioperative predictors were evaluated for postoperative AKI. Univariate and multivariate regression analyses were conducted to identify predictors associated with AKI following surgery. The primary end point was the incidence of postoperative AKI, while the secondary end points included in-hospital mortality and other major surgical complications.

**Results:**

This study enrolled 224 patients in all. There were 155 (69.2%) patients with postoperative AKI, including 55 (24.6%) with KDIGO stage 1, 45 (20.1%) with stage 2 and 55 (24.6%) with stage 3. Twenty-eight patients (12.5%) needed renal replacement therapy after surgery. The total in-hospital mortality was 2.7% (AKI vs. non-AKI: 3.2% vs. 1.4%, *p* = 0.669). Multivariate regression analysis found total protein concentrations [odds ratio (OR) 1.136, 95% confidence interval (CI): 1.032–1.250, *p* = 0.009], intraoperative blood loss (OR 1.002, 95% CI: 1.000–1.004, *p* = 0.042) and ventilation time (OR 1.011, 95% CI: 1.001–1.021, *p* = 0.026) were independently associated with AKI. The area under the receiver operating characteristic curve was 0.688 (95% CI: 0.617–0.759). Our predictive model demonstrated a sensitivity of 72.5% and a specificity of 57.4%. The ordinal logistic regression analysis found that age (OR 1.055, 95% CI: 1.027–1.084, *p* < 0.001), body mass index (OR 1.194, 95% CI: 1.104–1.291, *p* < 0.001), a high total protein to albumin ratio (OR 2.615, 95% CI: 1.234–5.540, *p* = 0.012) and ventilation time (OR 1.005, 95% CI: 1.001–1.008, *p* = 0.005) were independently associated with the severity of AKI.

**Conclusion:**

A higher preoperative total protein to albumin ratio independently predicted more severe postoperative AKI in patients undergoing surgical treatment for ATAAD. Monitoring preoperative total protein concentrations and the total protein to albumin ratio may assist in identifying patients at higher risk of progressing to severe AKI, though further multicenter validation is required.

## Introduction

Acute type A aortic dissection (ATAAD) is a serious emergency in the field of cardiovascular surgery, characterized by significant rates of morbidity and mortality. Total aortic arch replacement (TAAR) in conjunction with stent elephant implantation (SETI) is an effective approach for treating ATAAD. Cardiac surgery-related acute kidney injury (CS-AKI) is a frequent complication that patients may experience following cardiac procedures (1). The prevalence of AKI, according to the criteria established by Kidney Disease Improving Global Outcomes (KDIGO), following the repair of ATAAD has been reported to range from 50% to 70% (2–4). The KDIGO criteria comprise the most recent definition of AKI and are routinely applied in medical practice (5).

Previous studies have demonstrated that postoperative AKI was linked to a prolonged hospitalization, a higher likelihood of readmission, high in-hospital mortality, decreased long-term survival rate and increased burden for health care in patients following ATAAD repair (6, 7).

The pathophysiology of CS-AKI involves multiple factors (1). TAAR surgery can increase the probability of AKI because of the complexity of the surgery, deep hypothermic circulatory arrest (DHCA) and an extended cardiopulmonary bypass (CPB) duration. Currently, there are no targeted therapies for postoperative AKI, highlighting the importance of preventive measures. Identifying risk factors for AKI following ATAAD repair is crucial for the prevention of postoperative AKI and the improvement of patient outcomes. However, limited studies have reported on the prevalence and predictors for AKI, according to the KDIGO criteria, among patients undergoing surgery for ATAAD.

Serum total protein primarily comprises albumin and globulin. While hypoalbuminemia and hyperglobulinemia have individually been associated with AKI, the total protein to albumin ratio (TP-ALB ratio) integrates their combined effects, potentially serving as a stronger predictor of systemic inflammation and endothelial dysfunction (8, 9). Increasing evidence has shown that the serum TP-ALB ratio can be a prognostic marker reflecting both inflammatory and nutritional status in a variety of diseases such as septic AKI, which is prevalent among patients with critical illness (8). However, limited evidence is available for patients undergoing heart surgery.

Therefore, we designed a retrospective analysis to investigate the incidence, predictors, and short-term outcomes of AKI in patients undergoing surgery for ATAAD, using the KDIGO criteria.

## Methods

### Study design

This retrospective cohort analysis was conducted at Zhongshan Hospital, Fudan University, China. Ethical approval was obtained from the Ethics Committee of Zhongshan Hospital, Fudan University. Given the retrospective design, the need for individual informed consent was waived (ethical approval number: B2021-621). This study adhered to the rules of the Declaration of Helsinki (as revised in 2013) and principles of Good Clinical Practice.

### Patients

A retrospective review of medical records was conducted for patients who were diagnosed with ATAAD and underwent TAAR and SETI surgery from January 2020 to December 2021 at the Cardiac Surgery Center of Zhongshan Hospital. Patients who died intraoperatively or within 72 h postoperatively were excluded to focus on AKI as a primary outcome, as early mortality was often driven by factors unrelated to acute renal injury, such as catastrophic hemorrhage and acute cardiac failure. Patients who with incomplete baseline or postoperative serum creatinine (sCr) concentrations, and those who had undergone preoperative renal replacement therapy (RRT) for chronic kidney disease were also excluded. Following the review of electronic medical records, 224 patients were selected for the final analysis.

### Definitions

ATAAD was diagnosed by the thoracic and abdominal computed tomography angiography examination according to Stanford classification criteria for type A at our institution. The duration from symptom onset to hospital admission was less than 14 days. We referred to the European registry of type A aortic dissection definition criteria for definitions of clinical variables (10). Briefly, hypertension was defined as systemic arterial pressure exceeding 150/80 mmHg or the utilization of antihypertensives, based on the European registry of type A aortic dissection definition criteria, where stricter blood pressure control was recommended to reduce dissection progression risk (10). Prior stroke was defined as a neurological condition due to ischemia or hemorrhage that persisted for more than 24 h before surgery. Diabetes was defined as elevated blood glucose levels necessitating the use of oral medications or insulin therapy. Renal dissection was characterized by a computed tomography finding indicative of compromised renal perfusion. Paraplegia was characterized by bilateral limb weakness or multiple sensory disorders below the level of spinal cord injury. Major surgical complications were defined as postoperative RRT, stroke, postoperative paraplegia and in-hospital mortality.

### Data collection

Trained staff systematically collected patients’ clinical and laboratory values from their electronic medical records.

Preoperative variables that were evaluated comprised age, sex, height, weight, body mass index (BMI), drinking history, smoking history, the left ventricular ejection fraction, preoperative sCr concentrations, blood urea nitrogen concentrations, the estimated glomerular filtration rate (eGFR), total protein (TP) concentrations, albumin (ALB) concentrations, the duration from symptom onset to surgery, hemoglobin levels and comorbidities. The comorbidities consisted of diabetes mellitus, hypertension, previous stroke, previous cardiac surgery, aortic regurgitation, renal dissection, hemopericardium, coronary artery disease, Marfan syndrome and coronary angiography <72 h before surgery.

Intraoperative variables that were evaluated included the CPB duration, aortic cross-clamp duration, DHCA time, nasopharyngeal and rectal temperatures during circulatory arrest, intraoperative blood loss, administration of packed red blood cells and fresh frozen plasma, and the specific type of surgical intervention.

Postoperative variables that were evaluated included postoperative RRT, duration of intubation, hospital length of stay, intensive care unit (ICU) stay duration, and mortality during hospitalization.

### Criteria for diagnosing AKI

AKI was identified using the KDIGO criteria, primarily determined by comparing the baseline and peak sCr concentrations within 7 days following the surgery (5). The latest preoperative sCr value was regarded as the baseline sCr level. We applied the sCr criteria to diagnose AKI and did not include urine output because of its inaccuracy in retrospectively collected data.

Based on the KDIGO criteria, AKI was identified by a rise in sCr concentrations of at least 26.5 μmol/L over a period of 48 h or reaching to 1.5 times or more above the baseline within a week. Stage 1 AKI was diagnosed when the sCr concentrations were 1.5–1.9 times that at baseline, or showed a rise of at least 26.5 μmol/L. Stage 2 was diagnosed by a rise in sCr concentrations to 2.0–2.9 times the baseline. Stage 3 was diagnosed by a rise in sCr concentrations to threefold or greater than the baseline level, an absolute sCr rise of at least of 353.6 μmol/L, or the commencement of RRT. Primary indications for RRT were anuresis, volume overload and obvious biochemical abnormalities.

All individuals were then divided into two groups: the AKI group and the non-AKI group.

### Surgical techniques

A median sternotomy was performed in all patients with ATAAD who underwent TAAR and SETI in our center. The surgical technique has been described previously (11). Briefly, a stent elephant trunk was implanted into the descending aorta in combination with total aortic arch replacement using a four-branched vascular graft for aortic reconstruction. To perform CPB, arterial cannulation was typically placed into the right axillary artery and the femoral artery. During the DHCA, selective cerebral perfusion was achieved via the right axillary artery. Venous cannulation was placed in the right atrium. CPB flow rate was maintained within the range of 2.2–2.4 L/(min·m^2^). The target mean arterial pressure was 50–80 mmHg during CPB. For myocardial protection, del Nido cardioplegic solution was administered via direct antegrade infusion in patients with anatomically normal coronary arteries. In cases where aortic dissection involved the coronary ostia, we adopted a combined antegrade and retrograde cardioplegia delivery strategy to ensure adequate myocardial protection throughout the operation. In addition, surgery was usually performed with DHCA, whereas antegrade cerebral perfusion was utilized for protecting the brain. Cerebral oxygen level was continuously monitored throughout the surgery. DHCA was initiated once the nasopharyngeal temperature ranged from 22°C to 24°C, while the rectal temperature was within the range of 27°C to 28°C. The brain received a perfusion rate of 5–10 ml/kg/min following the initiation of selective antegrade cerebral perfusion. Rewarming was initiated immediately once the anastomosis with the left common carotid artery was completed. After surgeries, patients were transferred to ICU for postoperative management. Throughout the study period, no major alterations were observed in anesthesia, surgical techniques or perioperative management.

### Statistical analysis

All analyses were conducted utilizing the statistical software package R version 4.3.2 (available at https://www.r-project.org/). Continuous variables were shown as the mean ± standard deviation when the data followed a normal distribution, or as the median (interquartile range) when the data did not follow a normal distribution. Categorical variables were shown as frequencies and their corresponding percentages. The chi-square test or Fisher's exact test was employed to compare categorical variables. For the analysis of continuous variables, a two-sample Student's *t*-test was utilized when the data follow a normal distribution. In contrast, the Mann–Whitney *U*-test was utilized to compare variables that deviated from a normal distribution. The differences among the three AKI stages were determined utilizing a one-way analysis of variance test. Binary logistic regression analysis was utilized to perform both univariate and multivariate examinations, with the objective of identifying independent factors associated with AKI. When multivariate analyses were implemented, only candidate variables which had *p*-values less than 0.2 by the univariate analysis, along with potential predictive variables, were included. Receiver operating characteristic (ROC) curves were constructed to evaluate the predictive model derived from multivariate logistic regression analyses. The ideal cut-off was identified using Youden's index. Additionally, multiple ordinal logistic regression analyses were conducted, integrating variables with *p*-values less than 0.2 from the analysis of variance, to determine the independent predictors related to the severity of AKI. Based on prior studies reporting an AKI incidence of 50%–70% in ATAAD patients (2–4), a sample size of 224 was estimated to provide 80% power (α=0.05) to detect a 15% difference in AKI risk between groups, assuming an odds ratio of 1.5. In all analyses, a two-tailed *p*-value below 0.05 was regarded as indicating statistical significance.

## Results

### Patients’ characteristics

A total of 224 patients were enrolled in this study. There were 155 patients (69.2%) who developed AKI, with 55 patients (24.6%) classified as KDIGO stage 1, 45 patients (20.1%) in stage 2, and 55 patients (24.6%) in stage 3. The median age and BMI were 54 (20) years and 25.7 (4.8), respectively, and 166 (74.1%) were men. Comorbidities included hypertension (71.4%), Marfan syndrome (6.7%) and diabetes (3.6%). Preoperatively, the median sCr concentration was 79.5 (38) μmol/L, and the median eGFR was 81 (28) ml/min/1.73 m². A total of 126 (56.2%) patients had a high TP-ALB ratio. The participants were categorized into two groups (low TP-ALB ratio group and high TP-ALB ratio group) based on an optimal threshold of 1.628, which was determined by performing ROC curve analysis and maximizing the Youden index. The median operative median CPB duration was 177 (53.3) min. After the operation, 28 (12.5%) patients received RRT and there were 6 (2.7%) deaths in hospital.

Table 1 illustrates the patient characteristics based on the development of AKI and its three stages. Regarding preoperative variables, there were significant differences in hypertension, the time of onset of symptoms to the operation, sCr concentrations, the eGFR, lactic acid (Lac) concentrations, blood glucose concentrations, albumin concentrations and the white blood cell count between patients with who developed AKI and those who did not (*p* < 0.05). In terms of intraoperative factors, the CPB duration and the peak Lac concentrations were notably higher in the AKI group in comparison to the non-AKI group. Furthermore, individuals in the AKI group exhibited higher peak sCr concentrations, had a greater likelihood of needing RRT, and experienced longer ventilation time and ICU stays after surgery, in comparison to those without AKI.

**Table 1:** Characteristics of the patients in relation to the presence of AKI and different stages of AKI according to the KDIGO criteria.

Variables	No-AKI	All AKI	Stage 1	Stage 2	Stage 3	*P*-value^a^	*P*-value^b^
	(*N* = 69)	(*N* = 155)	(*N* = 55)	(*N* = 45)	(*N* = 55)		
Preoperative variables							
Age (year)	52.0 (19.0)	55.0 (18.5)	54.0 (23.5)	54.0 (19.0)	58.0 (17.5)	0.305	0.173
Male gender	47 (68.1%)	119 (76.8%)	40 (72.7%)	33 (73.3%)	46 (83.6%)	0.188	0.343
BMI (kg/m²)	24.8 (4.3)	26.1 (5.0)	26.0 (4.9)	26.0 (4.2)	26.5 (6.9)	**0**.**025**	**0**.**040**
Aortic regurgitation	24 (34.8%)	67 (43.2%)	26 (47.3%)	16 (35.6%)	25 (45.5%)	0.243	0.489
Previous cardiac surgery	3 (4.3%)	4 (2.6%)	2 (3.6%)	1 (2.2%)	1 (1.8%)	0.679	1.000
Hypertension	42 (60.9%)	118 (76.1%)	39 (70.9%)	33 (73.3%)	46 (83.6%)	**0**.**025**	0.246
Smoking	8 (11.6%)	23 (14.8%)	9 (16.4%)	5 (11.1%)	9 (16.4%)	0.676	0.748
Drinking	6 (8.7%)	7 (4.5%)	4 (7.3%)	1 (2.2%)	2 (3.6%)	0.227	0.625
Coronary artery disease	2 (2.9%)	6 (3.9%)	0 (0.0%)	2 (4.4%)	4 (7.3%)	1.000	0.115
Marfan syndrome	7 (10.1%)	8 (5.2%)	4 (7.3%)	4 (8.9%)	0 (0.0%)	0.245	0.073
Left ventricular ejection fraction (%)	64.0 (4.0)	64.0 (5.0)	64.0 (4.5)	65.0 (5.0)	64.0 (5.5)	0.508	0.796
Diabetes	5 (7.2%)	3 (1.9%)	1 (1.8%)	1 (2.2%)	1 (1.8%)	0.061	1.000
Previous stroke	5 (7.2%)	12 (7.7%)	2 (3.6%)	4 (8.9%)	6 (10.9%)	1.000	0.409
Renal dissection	2 (2.9%)	5 (3.2%)	1 (1.8%)	1 (2.2%)	3 (5.5%)	1.000	0.626
Coronary angiography <72 hours of OR	1 (1.4%)	2 (1.3%)	0 (0.0%)	1 (2.2%)	1 (1.8%)	1.000	0.747
Hemopericardium	32 (46.4%)	78 (50.3%)	29 (52.7%)	24 (53.3%)	25 (45.5%)	0.665	0.683
Onset to operation (h)	32.0 (69.0)	20.0 (20.5)	23.0 (23.0)	20.0 (23.0)	21.0 (15.5)	**0**.**014**	0.151
sCr (umol/L)	77.0 (33.0)	83.0 (39.5)	84.0 (38.5)	82.0 (29.0)	92.0 (61.5)	**0**.**029**	0.112
eGFR (ml/min/1.73 m²)	85.0 (29.0)	81.0 (29.0)	85.0 (31.0)	84.0 (29.0)	75.0 (23.5)	**0**.**035**	0.678
BUN (mmol/L)	6.3 (3.2)	7.0 (3.1)	7.0 (2.8)	6.5 (2.6)	7.4 (4.1)	0.106	0.621
Hgb (g/dl)	12.8 ± 1.6	13.2 ± 1.9	12.9 ± 1.8	13.5 ± 1.8	13.2 ± 2.0	0.160	0.305
Lac (mmol/L)	1.3 (0.9)	1.9 (2.1)	1.6 (1.5)	1.7 (2.0)	2.5 (2.1)	**<0**.**001**	0.154
Blood glucose(mmol/L)	6.9 (2.4)	7.9 (2.1)	7.6 (2.0)	7.5 (2.0)	8.3 (1.8)	**0**.**004**	**0**.**038**
ALT (U/L)	23.0 (18.0)	28.0 (26.0)	25.0 (15.5)	25.0 (28.0)	38.0 (35.0)	**0**.**038**	0.276
AST (U/L)	25.0 (17.0)	26.0 (22.5)	24.0 (19.0)	24.0 (17.0)	33.0 (28.0)	0.308	0.359
cTnT (ug/L)	0.015 (0.038)	0.019 (0.035)	0.016 (0.035)	0.015 (0.028)	0.023 (0.037)	0.430	0.509
BNP (pg/ml)	219.0 (297.1)	268.9 (432.8)	294.0 (432.5)	251.8 (359.7)	302.0 (509.8)	0.240	0.284
TP (g/L)	65.0 (8.0)	65.0 (6.0)	64.0 (6.0)	66.0 (5.0)	65.0 (8.5)	0.334	**0**.**040**
ALB (g/L)	39.0 (7.0)	40.0 (5.0)	39.0 (4.5)	41.0 (4.0)	39.0 (5.0)	0.343	0.367
High TP-ALB ratio	36 (52.2%)	90 (58.1%)	27 (49.1%)	28 (62.2%)	35 (63.6%)	0.467	0.263
WBC (10^9^/L)	10.7 (4.4)	12.7 (4.5)	12.7 (3.9)	12.7 (5.1)	12.6 (5.8)	**0**.**001**	0.639
PLT (10^9^/L)	160.0 (68.0)	152.0 (76.0)	170.0 (82.0)	155.0 (80.0)	148.0 (62.0)	0.572	0.239
Intraoperative variables							
CPB duration (min)	165.0 (39.0)	180.0 (51.5)	172.0 (44.0)	180.0 (58.0)	190.0 (60.0)	**0**.**036**	**0**.**005**
Aortic cross clamp time (min)	98.0 (36.0)	99.0 (40.0)	96.0 (28.0)	101.0 (47.0)	97.0 (45.5)	0.284	0.066
PPC time (min)	60.0 (27.0)	64.0 (26.0)	63.0 (25.5)	61.0 (21.0)	72.0 (30.0)	0.138	0.051
Circulatory arrest time (min)	19.0 (5.0)	18.0 (4.5)	19.0 (3.5)	17.0 (4.0)	19.0 (7.0)	0.698	0.098
Nasopharyngeal temperature at CA (°C)	22.5 (1.0)	22.4 (1.3)	22.5 (1.4)	22.5 (1.0)	22.3 (1.3)	0.947	0.668
Rectal temperature at CA (°C)	26.5 ± 1.1	26.4 ± 1.1	26.4 ± 1.2	26.3 ± 1.2	26.4 ± 1.1	0.534	0.700
Urine volume (ml)	200.0 (200.0)	200.0 (200.0)	200.0 (125.0)	200.0 (200.0)	200.0 (150.0)	0.162	0.531
Ultrafiltration (ml)	3,500.0 (1,000.0)	3,800.0 (1,350.0)	3,600.0 (1,250.0)	4,000.0 (1,000.0)	3,700.0 (1,500.0)	0.052	0.695
Intraoperative blood loss (ml)	600.0 (300.0)	700.0 (400.0)	700.0 (300.0)	600.0 (300.0)	800.0 (450.0)	0.071	**0**.**006**
Intraoperative amount of PRBC (units)	4.0 (6.0)	4.0 (6.0)	4.0 (6.0)	4.0 (6.0)	4.0 (4.0)	0.562	**0**.**040**
Intraoperative amount of plasma (ml)	600.0 (600.0)	600.0 (800.0)	600.0 (600.0)	600.0 (600.0)	600.0 (400.0)	0.353	**0**.**010**
Lowest Hgb (g/dl)	6.8 (1.6)	7.1 (1.6)	6.8 (1.6)	7.4 (1.8)	7.1 (1.5)	0.120	0.678
Peak Lac (mmol/L)	2.7 (1.8)	3.8 (2.4)	3.3 (1.7)	3.9 (2.4)	4.6 (3.6)	**<0**.**001**	**<0**.**001**
Peak blood glucose (mmol/L)	11.5 (3.1)	11.8 (2.4)	11.4 (2.4)	11.8 (2.7)	12.3 (1.9)	0.219	0.277
Operative procedures							
TAAR	51 (73.9%)	108 (69.7%)	46 (83.6%)	27 (60.0%)	35 (63.6%)	0.633	**0**.**016**
TAAR + Bentall	6 (8.7%)	16 (10.3%)	4 (7.3%)	5 (11.1%)	7 (12.7%)	0.811	0.714
TAAR + David	9 (13.0%)	16 (10.3%)	2 (3.6%)	9 (20.0%)	5 (9.1%)	0.646	**0**.**030**
TAAR + others	3 (4.3%)	15 (9.7%)	3 (5.5%)	4 (8.9%)	8 (14.5%)	0.286	0.283
Postoperative variables							
RRT	0 (0.0%)	28 (18.1%)	0 (0.0%)	0 (0.0%)	28 (50.9%)	**<0**.**001**	**<0**.**001**
Stroke	4 (5.8%)	23 (14.8%)	3 (5.5%)	7 (15.6%)	13 (23.6%)	0.074	**0**.**019**
Paraplegia	0 (0.0%)	2 (1.3%)	0 (0.0%)	1 (2.2%)	1 (1.8%)	1.000	0.747
Peak Cr (umol/L)	97.0 (36.0)	203.0 (172.0)	141.0 (65.5)	195.0 (98.0)	347.0 (186.5)	**<0**.**001**	**<0**.**001**
Ventilation time (h)	23.0 (19.0)	47.0 (86.5)	26.0 (35.5)	35.0 (44.0)	98.0 (161.0)	**<0**.**001**	**<0**.**001**
Length of ICU (days)	4.0 (3.0)	6.0 (6.0)	5.0 (3.0)	5.0 (4.0)	9.0 (12.0)	**<0**.**001**	**<0**.**001**
Length of in hospital (days)	13.0 (6.0)	13.0 (6.5)	12.0 (3.5)	13.0 (7.0)	15.0 (10.0)	0.549	**0**.**005**
In-hospital mortality	1 (1.4%)	5 (3.2%)	0 (0.0%)	0 (0.0%)	5 (9.1%)	0.669	**0**.**012**

Data are presented as the number (%) for categorial variables, mean ± standard deviation for continuous variables with a normal distribution and median (interquartile range) for continuous variables with a non-normal distribution. AKI, acute kidney injury; BMI, body mass index; sCr, serum creatinine; BUN, blood urea nitrogen, eGFR, estimated glomerular filtration rate; TP, total protein; ALB, albumin; ALT, alanine transaminase; AST, aspartate transaminase; Hgb, hemoglobin; TNT, troponin t; BNP, B-type natriuretic peptide; WBC, white blood cells; PLT, platelets; CPB, cardiopulmonary bypass; PRBCs, packed red blood cells; TAAR, total aortic arch replacement; CA, circulatory arrest; PPC, posterior parallel circulation; RRT, renal replacement therapy; ICU, intensive care unit; KDIGO, Kidney Disease Improving Global Outcomes. Bold values indicate statistical significance at *p* < 0.05.

^a^ Comparison between patients without AKI and those with all three stages of AKI.

^b^ Comparison between patients with AKI stages 1, stage 2, and stage 3.

There were significant differences in BMI, preoperative blood glucose and TP concentrations, intraoperative peak Lac concentrations, CPB duration, blood loss, amount of packed red blood cells and plasma, frequency of TAAR, frequency of TAAR + David, postoperative RRT requirement, incidence of stroke, peak sCr concentrations, and ventilation time among patients at three distinct stages (*p* < 0.05) (Table 1). Our analysis demonstrated that an increased in the severity of AKI was associated with a corresponding rise in the duration of ICU stays, days of hospitalization, and in-hospital mortality rate.

### Univariate analysis of risk factors for AKI

The results of univariate analysis identifying risk factors for AKI, are summarized in Table 2. Hypertension, the time of symptom onset to the operation, sCr concentrations, the eGFR, Lac concentrations, blood glucose concentrations, the white blood cell count, CPB duration, ultrafiltration, peak Lac concentrations and the ventilation time were all significantly related to the development of AKI.

**Table 2:** Univariate logistic regression analysis of risk factors for AKI.

Variables	Odds ratio	95% CI	*P*-value
Age	1.009	0.986–1.033	0.430
Male gender	1.547	0.825–2.902	0.174
BMI	1.073	0.998–1.153	0.056
Aortic regurgitation	1.428	0.792–2.572	0.236
Previous cardiac surgery	0.583	0.127–2.677	0.488
Hypertension	2.050	1.116–3.767	**0**.**021**
Smoking	1.329	0.562–3.139	0.517
Drinking	0.497	0.160–1.537	0.225
Coronary artery disease	1.349	0.265–6.858	0.718
Marfan syndrome	0.482	0.168–1.387	0.176
Left ventricular ejection fraction	0.972	0.899–1.052	0.482
Diabetes	0.253	0.059–1.089	0.065
Previous stroke	1.074	0.363–3.176	0.897
Renal dissection	1.117	0.211–5.902	0.897
Coronary angiography <72 hours of OR	0.889	0.079–9.970	0.924
Hemopericardium	1.171	0.663–2.068	0.586
Onset to operation	0.991	0.986–0.996	**<0**.**001**
sCr	1.012	1.002–1.021	**0**.**015**
eGFR	0.984	0.970–0.999	**0**.**038**
BUN	1.100	0.975–1.242	0.122
Hemoglobin	1.011	0.995–1.027	0.183
Lac	1.598	1.220–2.093	**0**.**001**
Blood glucose	1.232	1.045–1.454	**0**.**013**
ALT	0.100	0.998–1.002	0.969
AST	1.004	0.998–1.009	0.243
cTnT	0.514	0.042–6.300	0.603
BNP	1.000	1.000–1.001	0.288
TP	1.032	0.984–1.082	0.190
ALB	1.044	0.979–1.113	0.191
High TP-ALB ratio	1.269	0.718–2.244	0.412
WBC	1.167	1.063–1.282	**0**.**001**
PLT	0.998	0.994–1.003	0.428
CPB duration	1.009	1.002–1.017	**0**.**016**
Aortic cross clamp time	1.006	0.997–1.016	0.204
PPC time	1.012	0.998–1.025	0.085
Circulatory arrest time	0.987	0.937–1.040	0.634
Nasopharyngeal temperature at CA	1.057	0.806–1.385	0.690
Rectal temperature at CA	0.923	0.720–1.185	0.532
Urine volume	0.998	0.996–1.000	0.123
Ultrafiltration	1.000	1.000–1.001	**0**.**036**
Intraoperative blood loss	1.001	1.000–1.003	0.052
Intraoperative amount of PRBC	1.031	0.934–1.139	0.543
Intraoperative amount of plasma	1.000	0.999–1.001	0.630
Lowest Hgb	1.261	0.978–1.627	0.074
Peak Lac	1.352	1.134–1.612	**0**.**001**
Peak blood Glucose	1.063	0.937–1.205	0.342
TAAR	0.811	0.429–1.538	0.519
TAAR + Bentall	1.209	0.452–3.234	0.706
TAAR + David	0.767	0.321–1.833	0.551
TAAR + others	2.357	0.660–8.424	0.187
Ventilation time	1.015	1.006–1.023	**0**.**001**

AKI, acute kidney injury; BMI, body mass index; sCr, serum creatinine; BUN, blood urea nitrogen, eGFR, estimated glomerular filtration rate; TP, total protein; ALB, albumin; ALT, alanine transaminase; AST, aspartate transaminase; Hgb, hemoglobin; TNT, troponin t; BNP, B-type natriuretic peptide; WBC, white blood cells; PLT, platelets; CPB, cardiopulmonary bypass; PRBCs, packed red blood cells; TAAR, total aortic arch replacement; CA, circulatory arrest; PPC, posterior parallel circulation; CI, confidence interval. Bold values indicate statistical significance at *p* < 0.05.

### Multivariate analysis of independent risk factors for AKI

Table 3 shows the results of multivariate analysis. TP concentrations [odds ratio [OR] 1.136, 95% confidence interval [CI]: 1.032–1.250, *p* = 0.009], intraoperative blood loss (OR 1.002, 95% CI: 1.000–1.004, *p* = 0.042) and the ventilation time (OR 1.011, 95% CI: 1.001–1.021, *p* = 0.026) were independent predictors associated with AKI.

**Table 3:** Multivariate logistic regression analysis of independent risk factors for AKI.

Variables	Coefficient	Odds ratio	95% CI	*P*-value
Constant	−10.770	0.000	0.000–0.035	**0**.**004**
Male gender	0.792	2.208	0.819–5.952	0.118
BMI	0.018	1.018	0.929–1.116	0.698
Onset to operation	−0.006	0.994	0.988–1.001	0.075
sCr	0.002	1.002	0.986–1.018	0.814
eGFR	−0.002	0.998	0.971–1.026	0.904
BUN	−0.028	0.972	0.805–1.174	0.768
Hemoglobin	−0.010	0.991	0.962–1.020	0.530
Lac	0.274	1.316	0.934–1.854	0.117
Blood glucose	−0.012	0.988	0.798–1.223	0.911
BNP	0.001	1.001	1.000–1.001	0.107
TP	0.127	1.136	1.032–1.250	**0**.**009**
ALB	−0.058	0.944	0.832–1.072	0.372
WBC	0.024	1.025	0.901–1.166	0.710
CPB duration	0.006	1.006	0.995–1.018	0.275
PPC time	−0.005	0.995	0.976–1.013	0.569
Urine volume	−0.001	0.999	0.997–1.002	0.609
Ultrafiltration	0.000	1.000	1.000–1.001	0.271
Intraoperative blood loss	0.002	1.002	1.000–1.004	**0**.**042**
Lowest Hgb	0.238	1.269	0.833–1.932	0.267
Peak Lac	0.064	1.066	0.830–1.369	0.618
Ventilation time	0.011	1.011	1.001–1.021	**0**.**026**

BMI, body mass index; sCr, serum creatinine; eGFR, estimated glomerular filtration rate; BUN, blood urea nitrogen; TP, total protein; ALB, albumin; BNP, B-type natriuretic peptide; WBC, white blood cells; CPB, cardiopulmonary bypass; PPC, posterior parallel circulation; Hgb, hemoglobin; CI, confidence interval. Bold values indicate statistical significance at *p* < 0.05.

### Predictive model and ROC curve

**Figure 1:**
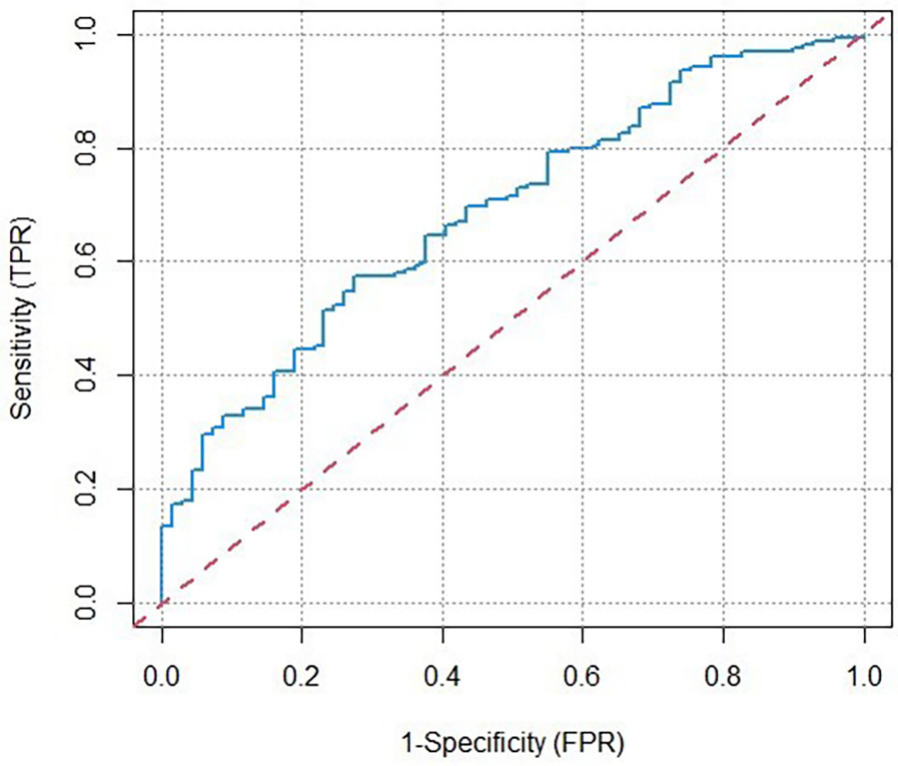
ROC curve of a predictive model of the occurrence of AKI. ROC, receiver operating characteristic; AKI, acute kidney injury; AUC, area under the curve.

The predictive model derived from the multivariate regression analysis was as follows: Y = 0.127 × TP + 0.002 × intraoperative blood loss + 0.011 × ventilation time–10.77, where Y denoted the odds ratio. The area under the ROC curve was 0.688, (95% CI: 0.617–0.759) (Figure 1). This model demonstrated a sensitivity of 72.5% and a specificity of 57.4%, using a threshold of 0.34, resulting in a maximum Youden index of 0.29.

### Ordinal logistic regression analysis of independent risk factors for the three stages of AKI

The results of ordinal regression analysis are presented in Table 4. Our analysis identified age (OR 1.055, 95% CI: 1.027–1.084, *p* < 0.05), BMI (OR 1.194, 95%, CI: 1.104–1.291, *p* < 0.05), a high TP-ALB ratio (OR 2.615, 95% CI: 1.234–5.540, *p* = 0.012), the TAAR + David procedure (OR 5.668, 95% CI: 1.359–23.646, *p* = 0.017) and ventilation duration (OR 1.005, 95%CI: 1.001–1.008, *p* = 0.005) as independent predictors of AKI severity.

**Table 4:** Multivariate ordinal logistic regression analysis of independent risk factors for the three stages of AKI.

Variables	Odds ratio	95% CI	*P*-value
Age	1.055	1.027–1.084	**<0**.**001**
BMI	1.194	1.104–1.291	**<0**.**001**
Coronary artery disease	0.913	0.113–7.385	0.932
Marfan syndrome	0.995	0.198–5.007	0.996
Onset to operation	0.999	0.991–1.006	0.780
sCr	0.995	0.985–1.005	0.331
Lac	0.960	0.769–1.199	0.719
Blood glucose	1.148	0.952–1.383	0.148
High TP-ALB ratio	2.615	1.234–5.540	**0**.**012**
CPB duration	1.016	0.993–1.039	0.167
Aortic cross clamp time	0.977	0.949–1.007	0.128
PPC time	0.994	0.967–1.022	0.665
Circulatory arrest time	1.081	0.994–1.176	0.070
Intraoperative blood loss	1.001	0.999–1.004	0.270
Intraoperative amount of PRBC	0.791	0.556–1.126	0.193
Intraoperative amount of plasma	1.002	0.999–1.005	0.126
Peak Lac	1.183	0.953–1.469	0.128
TAAR	0.377	0.139–1.022	0.055
TAAR + David	5.668	1.359–23.646	**0**.**017**
Ventilation time	1.005	1.001–1.008	**0**.**005**

AKI, acute kidney injury; BMI, body mass index; sCr, serum creatinine; TP, total protein; CPB, cardiopulmonary bypass; PRBCs, packed red blood cells; PPC, posterior parallel circulation; TAAR, total aortic arch replacement; ALB, albumin; CI, confidence interval. Bold values indicate statistical significance at *p* < 0.05.

## Discussion

In this study, we retrospectively analyzed the incidence of AKI based on KDIGO criteria, identified associated risk factors, developed a predictive model for AKI, and assessed the short-term prognosis in patients undergoing TAAR and SETI surgery for ATAAD.

Among all enrolled 224 patients, 155 (69.2%) developed postoperative AKI and 28 (12.5%) required postoperative RRT. In an international, prospective, multicenter clinical trial, 18.4% of patients experienced AKI after major surgeries, according to the KDIGO criteria (12). Based on the latest guidelines, the occurrence of AKI was approximately 30% in general cardiovascular surgeries and 5% required RRT (13, 14). In more complex aortic surgeries such as ATAAD repair, the incidence of AKI was higher, with the requirement for RRT ranged from 7% to 15% (14). The incidence of AKI in our study was comparable to that reported by Li et al. (71.94%) (4), but higher than that reported by two previous studies of patients with ATAAD who underwent surgery (53.0% and 50.4%) (2, 3). Similarly, Wang et al. found that 15.9% of patients who underwent ATAAD required postoperative RRT (3).

Our study revealed that the total postoperative in-hospital mortality rate was 2.7% (6/224) (AKI vs*.* non-AKI: 3.2% vs*.* 1.4%, *p* = 0.669). Nevertheless, Liu et al. found that patients undergoing ATAAD surgery who developed AKI exhibited a notably higher in-hospital mortality rate compared to those without AKI (13.1% vs. 1.9%; *p* = 0.025) (2). The exclusion of early deaths might account for the lower in-hospital mortality rate (2.7%) as compared to previous studies, which reported a 10%–15% mortality rate in ATAAD. Regarding the correlation between AKI and in-hospital mortality rate, we found no significant difference between these variables in this study. One potential explanation for the lack of significant findings may be the limited sample size, specially the small number of patients (*n* = 6) who died during their hospital stay. However, patients in the stage 3 group exhibited a significantly higher in-hospital mortality compared to those in stages 1 and 2.

In this study, we evaluated multiple potential risk factors to identify predictors of postoperative AKI. Univariate analysis showed that hypertension, the time of symptom onset to the operation, preoperative sCr concentrations, the eGFR, Lac concentrations, blood glucose concentrations, CPB duration, ultrafiltration volume, intraoperative peak Lac concentrations and postoperative ventilation duration were significantly related to the development of AKI. Our findings were largely in agreement with those of several prior studies (13). Chiba et al. reported that a hematocrit level below 21% during CPB was linked to postoperative renal insufficiency in 112 patients who underwent surgery for ATAAD (15). Just et al. (13) reported that patients who were admitted as emergencies had a higher incidence of AKI (*p* < 0.001), which was consisted with our results.

The pathophysiology of AKI after CPB has not been fully determined. CPB resulted in endothelial damage and systemic inflammation in patients with cardiac surgery, which contributed to the development of perioperative organ dysfunction (16). Several patient-related factors contribute to a higher risk of AKI following CPB, however, these factors are non-modifiable. However, certain procedure-related factors, such as intraoperative hemodilution, low flow perfusion, low pressure conditions and intraoperative peak Lac concentrations, could potentially be improved by optimal management of CPB. Timely treatment of hypotension, correction of anemia, and ensuring adequate oxygen supply are beneficial for renal protection (17). Brownlee et al. investigated the correlations between hematocrit levels during CPB and the incidence of postoperative AKI in patients who underwent aortic arch surgery with DHCA (18). They discovered that a higher hematocrit during CPB was related to a decreased incidence of acute renal failure following surgery. Scholz et al. proposed that reduced oxygen supply to the kidney was a common underlying factor in various etiologies of AKI (19). Intraoperative insufficient tissue perfusion and cellular oxygenation might be the leading causes of CS-AKI. Meanwhile, numerous guidelines have recommended the goal-directed perfusion as a strategy to prevent AKI (17, 20, 21).

The multivariate regression analysis showed preoperative TP, bleeding volume during the surgery, and ventilation time after the surgery were independent predictors of AKI. Our results were consistent with the findings reported in prior studies. Wang and colleagues found that the duration of postoperative ventilation and preoperative cystatin C concentrations were independent risk factors for postoperative AKI (3). Li et al. identified BMI, CPB duration, red blood cell transfusions, and low protein levels as independent predictors of postoperative AKI (*p* < 0.05) (4). Moreover, Wang et al. performed a meta-analysis to investigate the predictors associated with postoperative AKI in patients undergoing surgery for ATAAD, and they revealed that sepsis, higher BMI, and older age were independent predictors of AKI following surgery (22).

In this study, ordinal logistic regression analysis revealed that a higher preoperative serum TP-ALB ratio was an independent predictor significantly associated with increased severity of AKI across all three stages. Our finding has seldom been reported regarding the complication of AKI in patients after operation for ATAAD (4). Yin et al. retrospectively assessed the prognostic value of the TP-ALB ratio among 309 patients with septic AKI and they discovered that the TP-ALB ratio upon admission was an independent predictor of 30-day and 90-day mortality rates, potentially functioning as a simple and cost-effective prognostic indicator (9). Lai and colleagues assessed the relationship between ALB and globulin levels and mortality in a cohort of 554 patients receiving peritoneal dialysis, and they found that individuals with lower globulin and higher ALB levels exhibited the lowest all-cause mortality rates, while those with higher globulin and lower ALB levels demonstrated the highest mortality rates (23). Our results showed that in patients with ATAAD, a high preoperative TP-ALB ratio, which resulted from elevated TP concentrations and reduced ALB concentrations, was independently associated with increased severity of postoperative AKI. To the best of our knowledge, it is the first report to explore the correlation between TP-ALB ratio and the incidence of AKI following cardiac surgery. Elevated TP-ALB ratio may reflect hyperglobulinemia-driven inflammation and hypoalbuminemia-induced glycocalyx damage, both of which exacerbate renal dysfunction (8, 19). A higher preoperative TP-ALB ratio was defined as >1.628 in our results, derived from ROC analysis. This threshold may help identify patients at risk of more severe postoperative AKI in patients undergoing surgical treatment for ATAAD. Furthermore, our data indicated that monitoring preoperative TP concentrations and the TP-ALB ratio may assist in identifying patients at higher risk of progressing to severe AKI. For emergency ATAAD patients with elevated TP-ALB ratios, preoperative strategies, such as albumin infusion to correct hypoalbuminemia or corticosteroid-based anti-inflammatory therapies, may potentially reduce the risk of AKI. However, further clinical trials are warranted to validate the efficacy and safety of these interventions.

The association between lower protein concentrations and the increased risk of AKI has been previously documented in the literature (24). Findik and colleagues found that patients with lower ALB concentrations before surgery faced a higher risk of developing AKI following heart procedures (25). Moreover, Yang et al. demonstrated that baseline serum ALB concentrations in AKI patients were linked to their prognosis, with improved outcomes observed as ALB concentrations increased (26). The mechanism of this finding is still unclear. The researchers hypothesized that the endothelial glycocalyx was susceptible to rapid degradation in a low-protein environment (27). Therefore, maintaining preoperative normal serum ALB concentrations is crucial for protecting and restoring endothelial glycocalyx (28). Human ALB demonstrated its efficacy in protecting the endothelial glycocalyx and preserving microvascular integrity in several pre-clinical studies (27). However, it remains unknown whether the infusion of exogenous ALB *in vivo* improves prognosis. Lee and colleagues conducted a prospective, randomized, controlled study involving patients with ALB levels below 4.0 g/dl who were scheduled for off-pump coronary artery bypass grafting, and they found that infusion of 20% exogenous ALB prior to surgery lowered the incidence of postoperative AKI (control group vs*.* ALB: 25.7% vs*.*13.7%, *p* < 0.05), however, 30-day mortality or other major morbidities showed no significant difference (29). High-quality evidence is required to determine whether human ALB infusion preoperatively improves prognosis in individuals undergoing heart surgery (30, 31).

Timely recognition of high-risk patients with AKI through predictive models is helpful for clinicians to proactively implement preventive strategies, thereby reducing the risk of postoperative AKI (1). Demirjian et al. established a prediction model on basis of perioperative laboratory values and validated its ability to predict the occurrence of AKI following cardiac surgery (32). Perioperative laboratory values in their model included changes in sCr concentrations, bicarbonate levels, serum albumin, blood urea nitrogen, sodium, and potassium concentrations during the perioperative period. Grieshaber et al. combined urinary biomarkers with clinical risk scores to predict CS-AKI and they found that the sensitivity for detecting all stages AKI within 24 h was 38%, while the specificity was 81% at a cut-off value of 0.3 (33). Li et al. created a machine learning predictive model for CS-AKI on the basis of a randomized controlled trial and a dataset and they demonstrated that age, preoperative sCr concentrations, and blood urea nitrogen concentrations had a positive correlation with the incidence of CS-AKI (34). Additionally, preoperative platelet counts, blood pressure levels, serum albumin concentrations, and weight exhibited negative correlations with the incidence of CS-AKI.

We identified three independent predictors associated with postoperative AKI in patients who underwent TAAR and SETI procedures for ATAAD. The predictors included preoperative TP concentrations, intraoperative blood loss and postoperative ventilation time. Moreover, the ROC curve analysis demonstrated that the predictive model for postoperative AKI following ATAAD in this study exhibited relatively strong predictive performance, achieving a sensitivity of 72.5% and a specificity of 57.4%. The predictive power of our model was similar to the results from Ma et al. (35) and Zong et al. (36). Our findings warrant validation through larger-scale trials, and we expect the specificity and sensitivity to be further enhanced in subsequent studies.

## Limitations

There are some limitations to this study. First, it was a retrospective, single-center and observational study with few patients. Second, data regarding long term renal function outcomes were not available for analysis. Finally, the small number of in-hospital deaths limited the statistical power to detect associations between AKI and mortality, highlighting the need for larger multicenter studies.

## Conclusion

A higher preoperative total protein to albumin ratio independently predicted more severe postoperative AKI in patients undergoing surgical treatment for ATAAD. Monitoring preoperative TP concentrations and the TP-ALB ratio may assist in identifying patients at higher risk of progressing to severe AKI. Multicenter randomized, controlled trials are necessary to validate whether the implementation of this risk prediction model can improve patient prognosis in the future.

## Data Availability

The original contributions presented in the study are included in the article/Supplementary Material, further inquiries can be directed to the corresponding authors.
